# Supplementary material for: Survival outcomes and prognostic factors in children and adults with medulloblastoma from a Latin America country: A retrospective cohort
Source: PLoS One. 2025 Oct 27;20(10):e0333611. doi: 10.1371/journal.pone.0333611 (PMC12558496; doi:10.1371/journal.pone.0333611)
Supplement: S1 Table — (DOCX) [file pone.0333611.s002.docx]

**Supplementary Table 1.** Comparison of the Lansky, Karnofsky, and ECOG performance status according to the function level of pediatric, adolescents, and adult patients.

| Performance status | Lansky (Children) | Karnofsky (Adults/Teens) | ECOG (Adults/Teens) |
| --- | --- | --- | --- |
| 0 | 100 – Fully active, normal | 100 – Normal, no complaints | 0 – Fully active |
|  | 90 – Minor restrictions in play | 90 – Minor signs/symptoms |  |
| 1 | 80 – Active, tires more quickly | 80 – Normal activity with effort | 1 – Restricted in strenuous activity, ambulatory |
|  | 70 – Reduced play, sits quietly | 70 – Cares for self, unable to do active work |  |
| 2 | 60 – Up >50% of waking hours | 60 – Occasional assistance needed | 2 – Ambulatory >50%, unable to work |
|  | 50 – Lying down, gets up occasionally | 50 – Requires assistance, considerable care |  |
| 3 | 40 – Mainly in bed, still alert | 40 – Disabled, requires special care | 3 – Limited self-care, confined to bed >50% |
|  | 30 – Confined to bed, awake | 30 – Severely disabled |  |
| 4 | 20 – Very limited movement | 20 – Very sick, hospital required | 4 – Completely disabled |
|  | 10 – No play, does not get out of bed | 10 – Moribund |  |
| 5 | 0 – Dead | 0 – Dead | 5 – Dead |
